# Successful treatment of staghorn stones with flexible ureteroscopy and thulium fiber laser (TFL) lithotripsy: initial experience with 32 cases

**DOI:** 10.1007/s00240-024-01598-9

**Published:** 2024-06-27

**Authors:** Tawiz Gul, Mahmoud laymon, Maged Alrayashi, Mohamed Abdelkareem, Morshed Salah

**Affiliations:** 1 Urology Section, Surgery Department, Hazm Mebaireek general hospital, Hamad medical corporation, Doha, Qatar; 2 College of Medicine, Qatar University, Doha, Qatar; 3 Urology and Nephrology center, Mansoura University, Mansoura, Egypt

**Keywords:** Staghorn stones, Flexible ureteroscopy, Thulium fiber laser

## Abstract

**Purpose:**

To investigate the efficacy and safety of flexible ureteroscopy with thulium fiber laser lithotripsy for management of renal staghorn stones.

**Materials and Methods:**

Thirty-two patients with staghorn stones were recruited. Stone characteristics including: width, length, volume and density were analyzed. Ablation speed, laser efficacy and laser activity were recorded. The primary outcome was to assess stone free rate after the procedure using spiral CT scan.

**Results:**

The median stone volume was 7339 (3183–53838) mm^3^. Median operative and lasing time were 135 (70–200) and 117 (50–180) minutes, respectively. The mean total energy delivered was 63.9 ± 30 KJ with a median ablation speed of 1.3 (0.5–4.9) mm^3^/sec. Mean laser efficacy was 7.5 ± 3.6 Joules/mm^3^. A total of 12 complications occurred in 8 patients (25%). The median hospital stay was 7 (3.5–48) hours and 30 patients (93.7%) were discharged on the same day of surgery. After the first session, seventeen patients (53%) were stone free with no residual fragments while six (19%) patients had residuals £ 2 mm. Nine patients (28%) had residuals > 2 mm with median residual size of 4 (3–9) mm. A second intervention was required in 4 cases.The overall stone free rate after completion of treatment was 65.6%.

**Conclusion:**

Flexible ureteroscopy with thulium fiber laser lithotripsy is a safe and effective treatment option for staghorn stones with stone free rate comparable to standard PCNL with advantages of minimal morbidity, minimal blood loss and shorter hospital stay.

## Introduction

Staghorn stones are the most complex form of nephrolithiasis that represent a challenge to the endourologist as complete stone clearance with acceptable morbidity are the ultimate goals of adequate management [1]. Percutaneous nephrolithotomy (PNL) is considered the gold standard treatment of staghorn stones [2]. However, the procedure is associated with high grade complications like sepsis, severe bleeding and lengthy hospital stay. Furthermore, multiple percutaneous tracts, multiple treatment sessions and auxiliary procedures could be needed to achieve complete stone clearance [3, 4].

Over the last decade, flexible ureteroscopy (FURS) has been widely used for treatment of renal stones in the range of 10–20 mm with high stone free rate (SFR) and low morbidity [5]. Introduction of modern digital and small sized scopes, use of high-power lasers for lithotripsy have encouraged urologists to use FURS for management of renal stones larger than 25 mm [6]. The initial results were encouraging, but obviously multiple sessions were required to achieve high SFR comparable to PNL [7–9]. Thulium fiber laser (TFL) provides several advantages over Holmium: YAG (Ho: YAG) laser including: higher water absorption coefficient (4.5 times that of Ho: YAG) leading to higher stone ablation rate as laser energy will be absorbed by water containing cavities on the stone surface, less peak power resulting in less retropulsion and use of smaller fibers down to 50 μm allowing better scope deflection and better irrigation [10]. Furthermore, TFL has superior ergonomics compared to high power Ho: YAG being delivered via smaller and lighter devices, less electricity consumption, less noise and air cooling (fan) is sufficient. Based on these observations, we thought to investigate whether FURS with TFL lithotripsy could be a viable option for management of large and complex renal staghorn stones through a prospective study.

## Patients and methods

### Study population

**Fig. 1:**
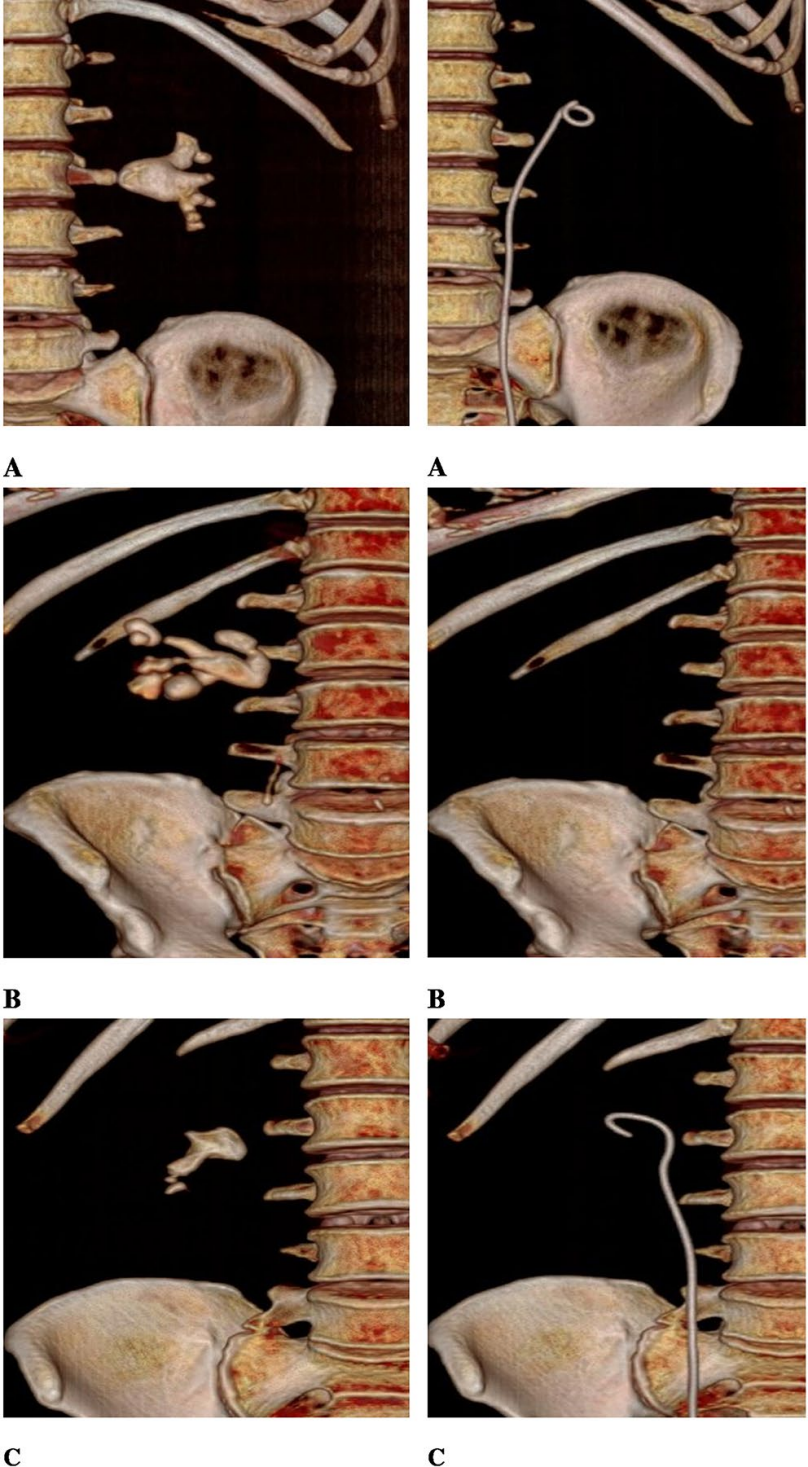
CT scan (3D) before and after treatment with flexible ureteroscopy and Thulium fiber laser lithotripsy for complete staghorn stones (**A**, **B**) and partial staghorn stone (**C**) with no residual fragment (RF)

**Fig. 2:**
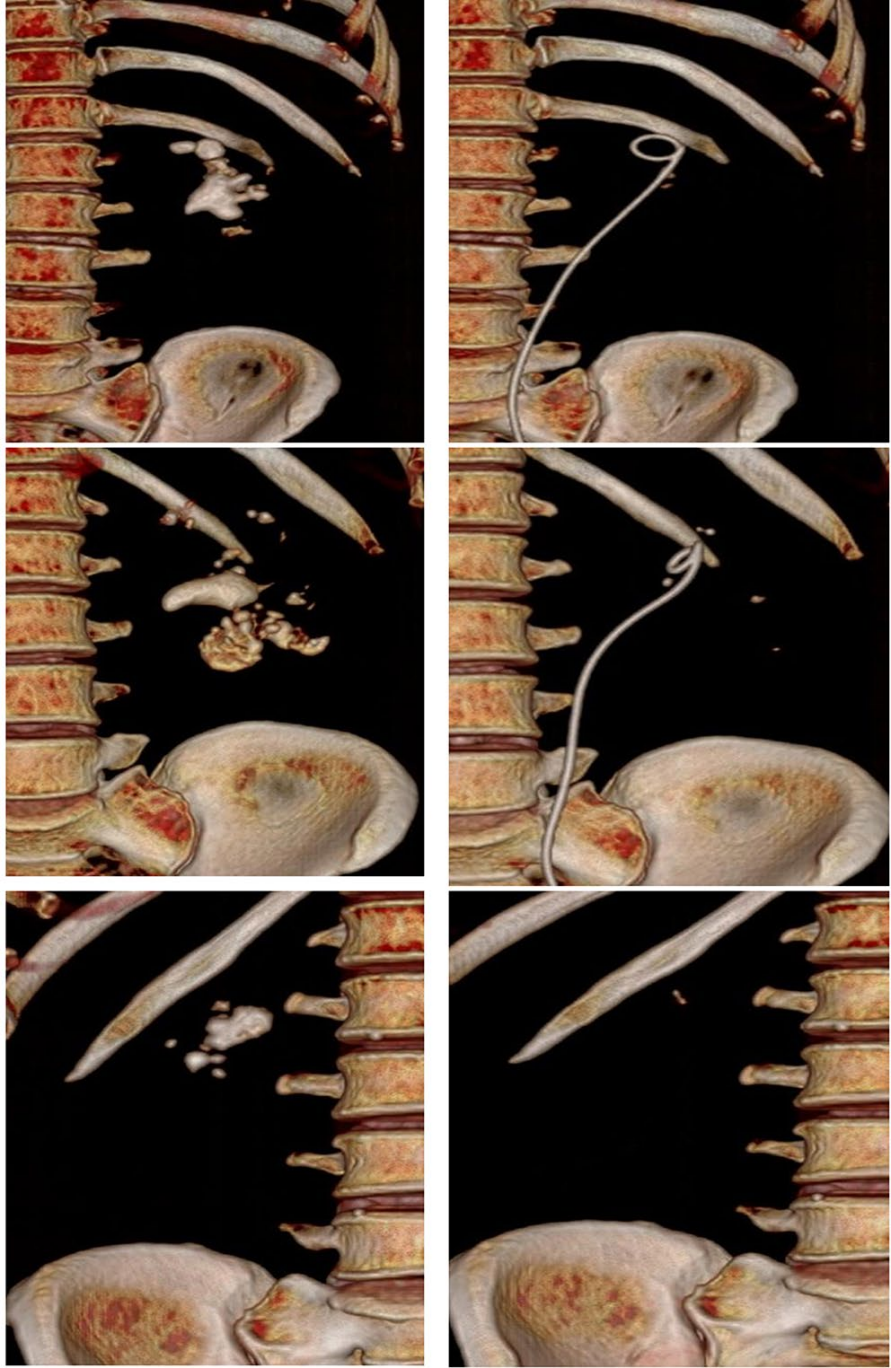
CT scan (3D) before and after treatment with flexible ureteroscopy and Thulium fiber laser lithotripsy with residual fragment (RF)

After institutional review board approval (IRB), we recruited patients who presented to our department with renal stones between February 1st 2023 till July 31st 2023. The inclusion criteria included patients with partial or complete staghorn stones. Partial staghorn stone was defined as renal pelvic stone branching into one or 2 calyces, while stones branching into the whole calyces were classified as complete staghorn stones (Figs. 1 and 2). Patients with congenital urinary tract anomalies, ureteric strictures and those who refused to participate were excluded. Eligible patients were asked to participate in the study after being fully informed about treatment options including the standard treatment by PNL. All patients signed an informed written consent in line with Good Clinical Practice and Declaration of Helsinki.

### Preoperative evaluation

Patient evaluation included detailed medical history, physical examination, body mass index (BMI), urinalysis, urine culture, complete blood count, serum biochemistry and coagulation profile. Estimated GFR was calculated according to modification of diet in renal disease (MDRD) formula. Patients with active urinary tract infection as evidenced by urine culture received antibiotics according to culture and sensitivity with documented negative culture before surgery. Preoperative imaging included: abdominal ultrasonography, X-ray KUB and computed tomography (CT) on abdomen and pelvis. Stone characteristics including, width, length, volume, shape and density were analyzed using the bone mode of the preoperative CT scan. Stone size was assessed in three dimensions: width, length in the axial cuts and height in the coronal cuts. The stone volume was calculated based on 3 dimensions in mm using the ellipsoid formula *(0.167x π x H x W x L).* Stone density was measured as average Hounsfield unit (HU) point value calculated with Synapse radiologic programme. 3D reconstruction of the images for all cases was performed by using imaging software (Volume Analyzer SYNAPSE VINCENT; FUJIFILM Corporation, Tokyo, Japan). Presence of calyceal stones separate from the main staghorn stone was documented and described as secondary calyceal stones.

### Surgical intervention

All procedures were performed under general anaesthesia with the patient placed in lithotomy position. Intravenous second generation Cephalosporins were administered to all patients with induction of anaesthesia according to the hospital policy. Initially, cystoscopy and a retrograde pyelography were done followed by semi-rigid ureteroscopy for inspection and dilatation of the ureter and exclude presence of stricture, stone fragments or tumors. Then a ureteral access sheath (UAS) is placed over a guide wire under fluoroscopic guidance. After introducing the scope into the UAS, maping and inspection of the renal pelvicalyceal system for localization of the stones was performed. A Single-Use Digital Flexible Ureteroscope WiScope® (OTU medical San Jose, CA, USA) with outer diameter of 8.6 Fr was utilized in all cases. The new TFL lithotripsy was performed by a 60-watt machine (Urolase SP, IPG Photonics, Russia) using a 200 μm laser fiber. The surgical technique involved a combination of laser dusting, fragmentation and pop corning of the stones. Several laser settings were utilized during lithotripsy depending on stone size, chemical composition and anatomical location. Stone clearance was assessed intraoperatively by direct visualization of the pelvicalyceal system. The goal was to obtain sub-millimeter fragments for spontaneous passage. A double-J was placed in all cases to be removed by outpatient cystoscopy within 1 to 2 months after surgery. A single expert surgeon (> 1000 FURS procedures) performed all cases (T.G).

## Operative parameters

All laser parameters were recorded including, pulse energy (Joules), pulse frequency (Hz), power (W), total laser energy (KJ) and laser time (minutes) as calculated by laser machine. Lithotripsy efficiency metrics included: ablation speed, laser efficacy and laser activity. Ablation speed was calculated by dividing stone volume by laser time (mm^3^/sec). Laser efficacy was calculated by dividing laser energy in joules by stone volume (J/mm^3^) [11]. Laser activity was calculated by dividing laser time to total operative time and reported as percentage (%).

### Postoperative care

All patients were kept in the postoperative care unit for monitoring of vital signs and urine output and those with uneventful postoperative course were discharged on the same day once full recovery was obtained. Patients were prescribed non-steroidal anti-inflammatory drugs (NSAIDS) for pain relief, anticholinergics for stent related symptoms, alpha-adrenergic blockers to facilitate passage of stone fragments and oral potassium citrate for chemolysis. All complications were recorded and stratified according to Dindo-Clavien system.

## Follow up

All patients were instructed to follow up within one week after surgery to assess the general condition then all patients were appointed to NCCT scan within 2–6 weeks after the procedure to accurately assess stone free rate (SFR) before stent removal. Renal dimercapto-succinic acid (DMSA) scan was done to all patients within 3 to 6 months after surgery to exclude renal scarring.

### Outcome measures

The primary outcome of the study was to assess SFR after the procedure using spiral CT scan. Residual stone fragments (RF) were classified into: zero fragments (Grade A), clinically insignificant RF ≤ 2 mm (Grade B) and sizable RF > 2 mm (Grade C). Patients with sizable residual fragments (> 2 mm) were further evaluated and counselled for an additional intervention according to stone size and location. Secondary outcomes included: postoperative complications, hospital stay and TFL efficiency measures.

### Statistical analysis

Continuous variables with normal distribution were expressed mean (± SD) and compared using t test while those with non-normal distribution were expressed as median (range). Categorical data were presented by number (%). The continuous variables were analyzed using Student’s t or Mann-Whitney *U* tests. Categorical data were analyzed by Chi-square. The strength of the relationship between variables was determined using Spearman’s correlation. Correlation strength was defined as very strong (*R* = 0.8–1), strong (*R* = 0.6–0.79), moderate (*R* = 0.4–0.59), weak (*R* = 0.2–0.39), and very weak (*R* = 0–0.19). Statistical significance was set at *p* < 0.05. The analysis was performed with the Statistical Package for Social Sciences, version 11.5 (SPSS, IBM, Armonk, NY).

## Results

### Patient and stone characteristics

A total of 32 consecutive patients were included in this study. Mean patient age was 41.7 ±9.3. Hypertension and diabetes mellitus were found in 13 (40.6%) and 9 (28%) patients, respectively with ASA score ≥ II seen in 16 patients. Twelve patients were obese with BMI > 30. Thirteen patients had recurrent stones and of them 6 patients had previous stone surgery. Median stone width and volume were 22.2 (19.2–32.8) mm and 7339 (3183–53,838) mm^3^, respectively. Mean stone density was 1004 ± 342 and 19 (59.4%) patients harbored stones with density > 1000 HU. Stone chemical composition was available for 23 patients and the most prevalent type was pure Calcium oxalate found in 10 patients. Detailed description of patient demographics and stone characteristics are shown in Table 1.

**Table 1:** Baseline characteristics of 32 patients with staghorn stones treated with Flexible ureteroscopy and TFL lithotripsy

Parameter	No (%)
Age (mean± SD)	41.7±9.3
Hypertension (yes)	13 (40.6%)
Diabetes Mellitus (yes)	9 (28%)
ASA Score (II, III)	16 (50%)
Body mass index (BMI) (kg/m^2^) (median, range)	27.6 (19–41)
Stone former (yes)	13 (40.6%)
Previous stone surgery (yes)	6 (18.75%)
Preoperative ureteric stent (yes)	8 (25%)
**Cause of preoperative stent**	
Tight ureter	6
Obstructive uropathy / pain	2
Preoperative serum creatinine (mg/dl) (median, range)	0.95 (0.8–1.08)
Preoperative eGFR (ml/min) (median, range)	87 (43–100)
Postoperative eGFR (ml/min) (median, range)	91.5 (49–100)
Preoperative Hb (gm/dl) (mean± SD)	14.4±1.5
Postoperative Hb (gm/dl) (mean± SD)	14.3±1.4
Hb deficit (gm/dl) (median, range)	0.2 (-1.0-1.3)
**Laterality**	
Right	13 (40.6%)
Left	19 (59.4%)
**Stone location**	
Renal pelvis	32 (100%)
Upper calyx	10 (31.3%)
Middle calyx	21 (65.6%)
Lower calyx	28 (87.5%)
**Stone Morphology**	
Partial staghorn (renal pelvis and 1 calyx)	10 (31.25%)
Partial staghorn (renal pelvis and 2 calyces)	14 (43.75%)
Complete staghorn	8 (25%)
Secondary calyceal stones (yes)	6 (18.75%)
Stone width (mm) (median, range)	22.2 (19.2–32.8)
Stone length (mm) (median, range)	20 (12–60)
Stone volume (mm^3^) (median, range)	7339 (3183–53,838)
Stone density (HU) (mean± SD)	1004 ± 342
**Stone density**	
< 1000	13 (40.6%)
≥ 1000	19 (59.4%)
**Chemical composition (available in 23 patients)**	
Calcium oxalate	10
Uric acid	6
Calcium phosphate	1
Calcium oxalate + uric acid	5
Calcium oxalate + Ca phosphate	1

### Operative findings

Median operative and lasing time were 135 (70–200) and 117 (50–180) minutes, respectively. The mean total energy delivered was 63.9±30 KJ with a median ablation speed of 1.3 (0.5–4.9) mm^3^/sec. Mean laser efficacy was 7.5 ±3.6 Joules/mm^3^. Laser was active during 85.7% (71-95%) of the total operative time Table 2.

**Table 2:** Perioperative outcomes of 32 patients with staghorn stones treated with RIRS and TFL lithotripsy

Parameter	No (%)
Operation time, minutes (median, range)	135 (70–200)
**Access sheath size (Fr)**	
10/12	19
11/12	8
12/14	5
**Lithotripsy techniques**	
Stone dusting	26 (81.3%)
Stone fragmentation	15 (46.8%)
Popcorning	28 (87.5%)
Stone extraction by basket	4 (12.5%)
Laser time (minutes) (median, range)	117 (50–180)
Pulse energy (Joules) (median, range)	0.5 (0.2–0.7)
Pulse frequency (Hz) (median, range)	20 (12–60)
Total energy used (KJ) (mean ± SD)	63.9 ± 30
Laser efficacy (J/mm^3^)	7.5 ± 3.6
Laser active time (%)	85.7% (71-95%)
Ablation speed (mm^3^/s) (median, range)	1.3 (0.5–4.9)
Length of hospital stay, hours (median, range)	7 (3.5–48)
Day case rate (same day discharge)	30 (93.7%)
Post-operative stent dwelling time, weeks (median, range)	4 (1–6)
**Initial stone free rate (SFR)**	
Grade A (no fragments)	17 (53.12%)
Grade B (RF ≤ 2 mm)	6 (18.75%)
Grade C (RF > 2 mm)	9 (28.12%)
Final stone free rate (SFR)	21 (65.6%)
**Reintervention for residual stones**	4 (12.5%)
RIRS	3
Semirigid ureteroscopy	1
Number of procedures /patient	1.12
**Overall complication rate**	8 (25%)
Flank pain (Grade I)	7
Hematuria (Grade I)	2
Vomiting (Grade I)	1
Febrile UTI (Grade II)	2

**Fig. 3:**
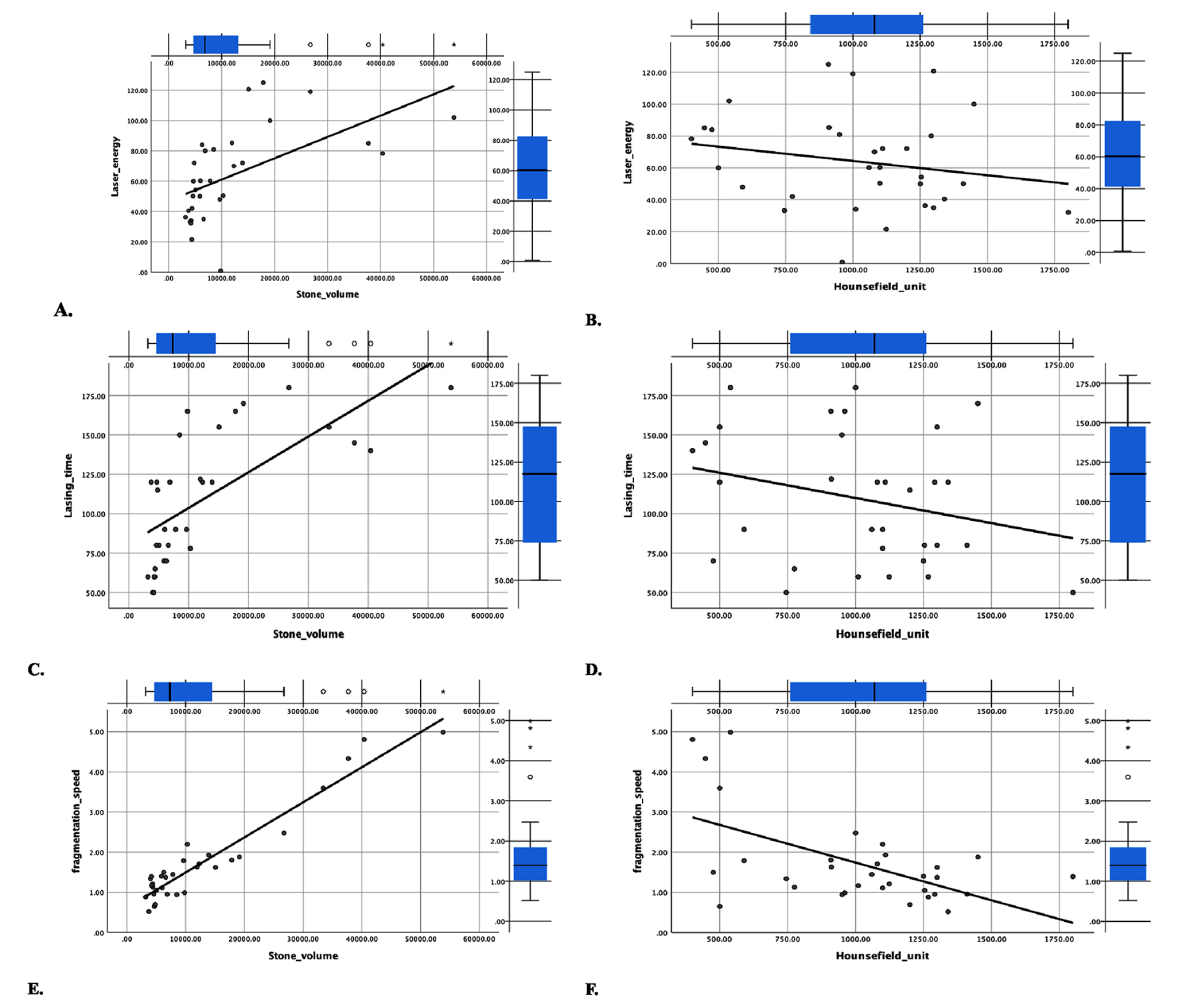
Relationship of laser energy with stone volume and density (**A**, **B**), relationship of laser time with stone volume and stone density (**C**, **D**) and relationship of ablation speed with stone volume and density (**E**, **F**)

Our study showed a strong positive correlation between stone volume and both laser time (*r* = 0.8, *p* < 0.0001) and fragmentation speed (*r* = 0.86, *p* < 0.0001) while a moderate negative correlation between stone volume and laser efficacy (*r* = − 0.6, *p* < 0.0001). In contrast, laser time (*r*= -0.24, *p* = 0.1), and laser efficacy (*r* = 0.26, *p* = 0.1) had weak relationship to stone density. A moderate negative correlation between stone density and ablation speed was encountered (*r* = -0.4, *p* = 0.023). Laser energy was strongly correlated with stone volume (*r* = 0.75, *p* < 0.0001) and weakly correlated to stone density (*r*= -0.24, *p* = 0.19) Fig. 3.

A total of 12 complications occurred in eight patients (25%). The median length of hospital stay was 7 (3.5–48) hours and 30 patients (93.7%) were discharged on the same day of surgery. Two patients developed mild hematuria and were managed conservatively. The median hemoglobin deficit was 0.2 (0-1.3) gm/dl and no single patient received packed RBCs transfusion. Two patients were admitted because of postoperative fever. Perioperative characteristics are detailed in Table 2.

### Stone free rate

After the first session, seventeen patients (53%) were stone free with no RF (Grade A) while six (18.75%) patients had RF≤ 2 mm (Grade B). Nine patients (28%) had RF > 2 mm (Grade C) with median RF size of 4 (3–9) mm (Figs. 1 and 2). A second intervention was required in 4 cases only, 3 underwent a second FURS procedure and a single patient underwent semirigid ureteroscopy for ureteric stone (Table 2). The overall SFR after the second intervention was 65.6%. Renal DMSA scan was available for 13 patients, and no cases of cortical scarring were detected.

## Discussion

The European association of urology (EAU) guidelines recommended PNL as the first line treatment option for staghorn stones as it provides adequately sized tract to the kidney to retrieve large stone fragments with less morbidity and faster recovery when compared to open surgery. Nonetheless, in the setting of staghorn calculi, PNL is associated with high rate of high-grade complications including sepsis, severe bleeding necessitating blood transfusion, injury to the surrounding organs. In addition, multiple tracts and auxiliary procedures are usually required which is associated with increased morbidity [12]. Recently, there has been an emerging role of FURS with laser lithotripsy in treatment of large size renal sones because of its less invasive nature compared to PNL and utilization of high-power lasers with different pulse modulation that allow dusting of the stones into fragments without need of basketing or retrieval of the stone fragments. A recent meta-analysis evaluating outcomes of FURS for renal stones > 2 cm, the SFR ranged between 77 and 96.7% after completion of treatment, with an average of 1.6 procedures per patient [6].

In this study we investigated the feasibility, safety and efficacy of FURS with TFL lithotripsy for treating partial and complete staghorn stones with a median stone volume of 7339 mm^3^ and the initial results were very promising. On one hand, the true SFR (no RF) was 53% after the initial treatment which is close to that reported after standard PNL that ranged between 49 and 56% [3, 4, 13]. Taking into consideration that spiral CT was used in all cases to assess SFR. Park et al. found that X-ray and CT controlled SFRs can differ dramatically (62.3 vs. 20.8%) [14]. We considered only patients with zero fragments to be stone free excluding those with insignificant RF < 2 mm [15].

On the other hand, the rate of postoperative complications was 25% and all were of minor grades (Grade I, II). It’s noteworthy that median hemoglobin deficit was 0.2 gm/dl and no patient received blood transfusion. In addition, 93% of cases were discharged home at the same day of surgery and only 2 cases were admitted because of febrile UTI and were treated with antibiotics. The overall complication rate after PNL for staghorn stones ranged between 18 and 32%, blood transfusion was required in 6–16% of cases while high grade complications (Grade III-V) ranged between 8 and 10% and the median length of hospital stay ranged between 2 and 6 days in recently published series [3, 4, 13, 16, 17].

Thulium fiber laser (TFL) has recently been introduced in our endourological armamentarium provoking a great interest because of its potential advantages over the standard Ho: YAG laser in terms of higher absorption coefficient, less retropulsion, the ability to work at a very high frequency with low energy levels resulting in very efficient dusting and shorter operative time [3, 10, 18, 19]. It has been observed that ablation speed for TFL is 2 to 5 times higher than Ho: YAG even with similar pulse energy and frequency settings [20]. High frequency modes result in higher ablation speed and TFL can attain higher frequency of up to 1000 Hz while keeping energy low [21]. TFL produces uniform pulse energy similar to that of Moses technology resulting in formation of bubbles within a single laser pulse typically smaller than Ho: YAG leading to minimal retropulsion [10, 22].

In this study, we evaluated the performance of TFL lithotripsy by the analysis of laser efficacy (energy needed to ablate 1 mm3 of stone volume, Joules/mm3) and ablation speed (the stone volume divided by the laser active time, mm3/s) as a surrogate of lithotripsy efficacy [11]. We found that use of TFL for effective treatment of staghorn stones, with a mean volume of 7339 mm^3^, required a mean 7.5 J/mm^3^ of stone volume with average ablation speed of 1.3 mm^3^/sec and active laser emission 85% of the total operative time. In addition, a strong positive correlation between stone volume and ablation speed (*r* = 0.86) and a moderate negative correlation between stone volume and laser efficacy (*r* = − 0.6) so the higher the stone volume, the less energy required for ablation (lesser J/mm3 required). In contrast, stone density had weak correlation with laser time and laser energy denoting the efficacy of TFL lithotripsy even in hard stones. These results were consistent with previously published studies evaluating performance of TFL in large stones [23]. Enikeev et al. also noted no correlation between laser time and stone density while using TFL during PNL [18].

Regarding Ho: YAG laser, Ventimiglia et al. reported a median laser efficacy of 19 J/mm3 and ablation speed of 0.7 mm3/s found that for a median stone volume of 1599 mm3 using 35 W machine [11]. Majdalany et al. assessed efficiency measures for Ho: YAG with Moses technology and found that for a mean stone volume of 290 mm3, mean laser efficacy was 38.2 J/mm3 and the mean ablation speed was 0.9 mm3/s [24]. In a RCT comparing Ho: YAG and TFL during mini-PNL, the authors noted shorter stone fragmentation time and shorter operative time in favour of TFL [25].

Another issue that is usually blown out is the higher temperature rise with the use of TFL because of higher absorption of TFL energy. Taratkin et al. compared thermal effects between TFL and Ho: YAG laser in an in vitro model by measuring water temperature. Energy settings for both lasers were adjusted at 0.2 J and 40 Hz with laser firing for 60 s and different irrigation rates were used. The authors documented that no significant difference in temperature rise between both lasers [26]. Theoretically the increased heat production may cause thermal injury to the renal parenchyma especially with prolonged use. In our study, there was no significant change in the estimated GFR before and after the procedure. In addition, renal DMSA scan that was available to 13 patients didn’t demonstrate any cortical scarring or areas of reduced perfusion after the procedure denoting the safety of utilization of TFL for prolonged time to disintegrate large volume renal stones.

To the best of our knowledge, this is the first prospective study documenting feasibility, efficacy and safety of FURS with TFL lithotripsy for treating exclusively staghorn stones with high success rate and minimal morbidity. Moreover, the vast majority of cases (93%) were discharged home safely at the same day of surgery with significantly less morbidity and shorter hospital stay. We also, reported efficiency measures of TFL to provide an arm for comparison of different laser types in future studies. Our study isn’t devoid of limitations, first is the small sample size but we report our initial experience and the feasibility to effectively manage large complex stones with TFL. Second, lack of comparison and randomization between FURS and the gold standard PNL for management of staghorn stones, however this issue should be discussed in future multicenter RCT. Lastly, long-term assessment of renal function wasn’t done to investigate whether the thermal effect of TFL especially when used for long time will affect renal function or not.

## Conclusion

Flexible ureteroscopy with new TFL lithotripsy is a safe and effective treatment option for renal staghorn stones with high SFR. The procedure is associated with minimal morbidity, minimal blood loss and shorter hospital stay. Prospective randomized controlled trials comparing FURS with TFL and PNL are highly indicated.

## Data Availability

No datasets were generated or analysed during the current study.

## Declarations

### Brief description

Flexible ureteroscopy & thulium fiber laser for management of staghorn stones.
