# Correction to Successful treatment of staghorn stones with flexible ureteroscopy and thulium fiber laser (TFL) lithotripsy: initial experience with 32 cases

**DOI:** 10.1007/s00240-024-01612-0

**Published:** 2024-09-06

**Authors:** Tawiz Gul, Mahmoud laymon, Maged Alrayashi, Mohamed Abdelkareem, Morshed Salah

**Affiliations:** 1 Urology Section, Surgery Department, Hazm Mebaireek general hospital, Hamad medical corporation, Doha, Qatar; 2 College of Medicine, Qatar University, Doha, Qatar; 3 Urology and Nephrology center, Mansoura University, Mansoura, Egypt

## Abstract

**Purpose:**

To investigate the efficacy and safety of flexible ureteroscopy with thulium fiber laser lithotripsy for management of renal staghorn stones.

**Materials and methods:**

Thirty-two patients with staghorn stones were recruited. Stone characteristics including: width, length, volume and density were analyzed. Ablation speed, laser efficacy and laser activity were recorded. The primary outcome was to assess stone free rate after the procedure using spiral CT scan.

**Results:**

The median stone volume was 7339 (3183–53838) mm^3^. Median operative and lasing time were 135 (70–200) and 117 (50–180) minutes, respectively. The mean total energy delivered was 63.9±30 KJ with a median ablation speed of 1.3 (0.5–4.9) mm^3^/sec. Mean laser efficacy was 7.5 ±3.6 Joules/mm^3^. A total of 12 complications occurred in 8 patients (25%). The median hospital stay was 7 (3.5–48) hours and 30 patients (93.7%) were discharged on the same day of surgery. After the first session, seventeen patients (53%) were stone free with no residual fragments while six (19%) patients had residuals ≤ 2 mm. Nine patients (28%) had residuals > 2 mm with median residual size of 4 (3–9) mm. A second intervention was required in 4 cases.The overall stone free rate after completion of treatment was 65.6%.

**Conclusion:**

Flexible ureteroscopy with thulium fiber laser lithotripsy is a safe and effective treatment option for staghorn stones with stone free rate comparable to standard PCNL with advantages of minimal morbidity, minimal blood loss and shorter hospital stay.

**Urolithiasis (2024) 52:102**

10.1007/s00240-024-01598-9

In the original version of this article, the abstract was missing and should have read.

The original article has been corrected.

